# How Does the Past of a Soccer Match Influence Its Future? Concepts and Statistical Analysis

**DOI:** 10.1371/journal.pone.0047678

**Published:** 2012-11-30

**Authors:** Andreas Heuer, Oliver Rubner

**Affiliations:** 1 Institute of Physical Chemistry, WWU Muenster, Muenster, Germany; 2 Center of Nonlinear Science (CeNoS), WWU Muenster, Muenster, Germany; Universidad Carlos III de Madrid, Spain

## Abstract

Scoring goals in a soccer match can be interpreted as a stochastic process. In the most simple description of a soccer match one assumes that scoring goals follows from independent rate processes of both teams. This would imply simple Poissonian and Markovian behavior. Deviations from this behavior would imply that the previous course of the match has an impact on the present match behavior. Here a general framework for the identification of deviations from this behavior is presented. For this endeavor it is essential to formulate an a priori estimate of the expected number of goals per team in a specific match. This can be done based on our previous work on the estimation of team strengths. Furthermore, the well-known general increase of the number of the goals in the course of a soccer match has to be removed by appropriate normalization. In general, three different types of deviations from a simple rate process can exist. First, the goal rate may depend on the exact time of the previous goals. Second, it may be influenced by the time passed since the previous goal and, third, it may reflect the present score. We show that the Poissonian scenario is fulfilled quite well for the German Bundesliga. However, a detailed analysis reveals significant deviations for the second and third aspect. Dramatic effects are observed if the away team leads by one or two goals in the final part of the match. This analysis allows one to identify generic features about soccer matches and to learn about the hidden complexities behind scoring goals. Among others the reason for the fact that the number of draws is larger than statistically expected can be identified.

## Introduction

How the past determines the future is naturally an important question which, however, in most cases is difficult to answer due to the complexity of the real world. This is different in the field of sports, where many aspects can be captured by well-defined numbers (such as goals in the case of soccer). Therefore this field is amenable to this question. In recent years researchers from the physics community have started to apply physics-oriented approaches to problems from the area of sports and in particular of soccer [1]–[3]. Specific examples for a quantitative analysis of the outcome of sports events can be found, e.g., in [4]–[8] and new ranking schemes have been proposed [9]. At first one might think that it is hard to find systematic laws to characterize such complex phenomena as soccer matches. One key step in this endeavor is the definition of appropriate observables to capture some key properties. In recent years we have concentrated on the formal characterization of the notion of a team strength and its practical determination [10]. In this way it was possible to ask questions about the variation of the team strength during a season [11] or the impact of a coach dismissal on the team strength [12]. Alternative concepts of team strengths have been studied, e.g., in Ref.[13] for the case of baseball.

Already a long time it has been realized that the distribution of goals, scored by a team, can be roughly described by a Poisson distribution [14]–[16]. Such a distribution is to be expected if the probability to score a goal in the next minute is constant within the whole match. In the most simple stochastic model of a soccer match one might simply assume that both teams score goals according to *independent* Poisson distributions. Closer inspection of the empirical goal distribution displays, however, some broadening as compared to a Poisson distribution. To rationalize this observation a model has been presented which postulates an increase of the goal rate with an increasing lead [7], [8]. This self-affirmative effect could indeed reproduce the fat tails in the empirical goal distribution. In later work it has been shown that at least for the German soccer league (Bundesliga) these fat tails just follow from the distribution of team strengths [11]. Therefore the fat tails do not contradict the notion that in an individual match the scoring of goals follows Poisson statistics without self-affirmative effects.

Interestingly, it turns out that the number of draws is significantly larger (approx. 10%) than expected from the assumption of independent Poisson distributions[16]. Different scenarios may lead to this effect. Here are two extreme cases: (1) A draw in the, let's say, 70th minute reduces the attempts of both teams to score another goal. This leads to an increased probability to keep this score. (2) A score of, e.g., 1∶0, may strongly enhance the willingness of the trailing team to score a goal to reach at least a draw. Whether or not any of these scenarios indeed explain the excess of draws is not clear a priori. Knowledge of such effects would allow one to gain information about psychological effects within a soccer match. The central aim of this work is to derive a stochastic description of the course of a soccer match without resorting to any ad hoc models. Recently, somewhat related questions have been analyzed, e.g., for the case of basketball [17] and tennis [18]. These results can then be compared with the present analysis.

The structure of this paper is as follows. In Sect.2 we discuss the statistical framework to elucidate the basic complexities of a soccer match. In Sect.3 the results of this analysis are presented which are finally discussed in Sect.4. As a data basis we take the matches from the Premier German soccer league (Bundesliga) between seasons 1968/69 and 2010/11 (excluding 1992/93 because for this single season the number of teams was different).

## Statistical Framework

In a specific match of team A vs. team B one may estimate the number of expected goals 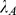 of team A and 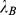 of team B based on the strength of both teams [16], [19], [20]. Here we choose the approach as used in Ref. [11]. In more detail, by taking the goal difference and the sum of all goals for the 33 other matches of both teams, considering the regression towards the mean, and adding a team-independent home advantage (see Ref.[10]) one can indeed obtain good estimates of 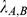. In what follows we define the goal rate as the probability to score a goal in the next minute. If the goal rate of, e.g., team A does not change during the match one can define the goal rate 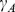 via 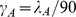. Note that a soccer match lasts for 90 minutes.

It is known that in the second half of a soccer match significantly more goals are scored than in the first half (57% vs. 43%). Thus, one may expect that the goal rate 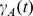 increases with time. To capture this effect more quantitatively, we introduce 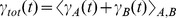 as the goal rate in minute 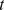, averaged over all matches, i.e. over all pairs 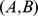. Note that 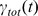 takes into account the goals of both teams.

**Figure 1:**
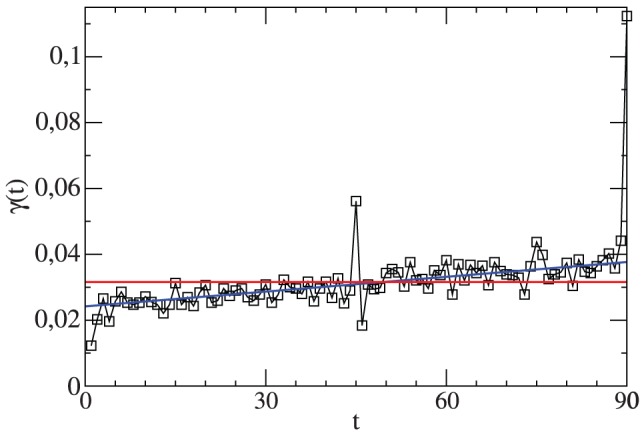
The average number of goals per minute in a match as a function of the time t. A more detailed interpretation of the data can be found in the subsequent section.

The resulting curve for 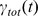 is shown in Fig.1. One can indeed see the general increase of 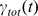 with time. Some additional specific features of Fig.1 will be discussed in Sect. III. When summing up 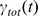 over all 90 minutes one obtains the total number of goals per match, denoted 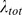. One finds 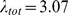. Correspondingly, a single team on average scores 1.53 goals per match.

For the time being we assume that it is indeed possible to define and determine a goal rate 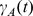 of team A at a given minute in an *individual* match. In reality this is impossible because playing soccer is much more complex than throwing a dice. The arrangement of the soccer players and the ball at some moment and possibly all other available pieces of information only allows a very rough estimation of the probability of a goal during the next minute. However, for the results of this work it will be sufficient to consider averages over a large number of appropriately selected matches. Therefore, in practice the average goal rate will be simply determined from counting the matches where a goal was scored in the minute under consideration.

For future purposes we introduce the normalized rate
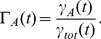
(1)


We remind again that the nominator contains the rate for an individual match whereas the denominator expresses the average over all matches. In any event 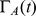 reflects the real course of the match. Furthermore we use the normalized expected number of goals in a specific match of team A
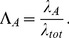
(2)


In contrast to Eq.1 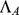 expresses the a priori expectation.

In general, the function 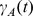 can be very complicated and can vary from match to match. For the future discussion it is helpful to identify a limit of maximum simplicity which we denote as the Poisson expectation. It can be formulated via two conditions: (1) The integral of the goal rate 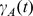 over the whole match is identical to the expected number of goals 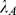 of team A in this match. (2) The time-dependence of 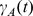 is, apart from a proportionality factor, identical to that of 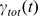 and thus follows the average behavior as shown in Fig.1. As a consequence one would have
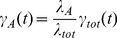
(3)which is equivalent to
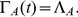
(4)


For this simple Poisson expectation the actual normalized goal rate 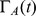 for a specific match thus equals the pre-match expectation 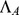.

The key goal of this work is to identify situations where Eq.4 and thus Eq.3 are not valid. For example we consider all matches for which the home team A just before minute 80 leads by m = 3 goals and ask for the probability that the home team scores a goal in the next minute. In what follows 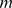 always denotes the goal difference. Then we define 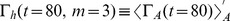. The prime indicates a conditional average. In this specific case we average the normalized goal rate of the home team over all matches for which the home team fulfills the required condition (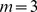 at minute 80). The actual calculation of 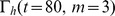 basically boils down to the calculation of 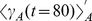 which, according to our conditioning, denotes the fraction of matches with 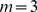 at minute 80 for which the home team scores a goal in minute 80. In analogy, we define 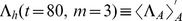 as the corresponding expectation value of 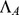 for the same subset of teams. Deviations from the relation 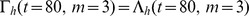 and the corresponding violation of Eq.4 directly imply that with a home lead of three goals in minute 80 the match behavior is different as expected from the simple Poisson expectation. As will be shown in this work for a home lead by three goals the probability of increasing the home lead is smaller than expected from the Poisson expectation. We note in passing that without conditioning, i.e. by averaging over all matches and all teams, one obtains by definition 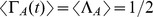.

**Figure 2:**
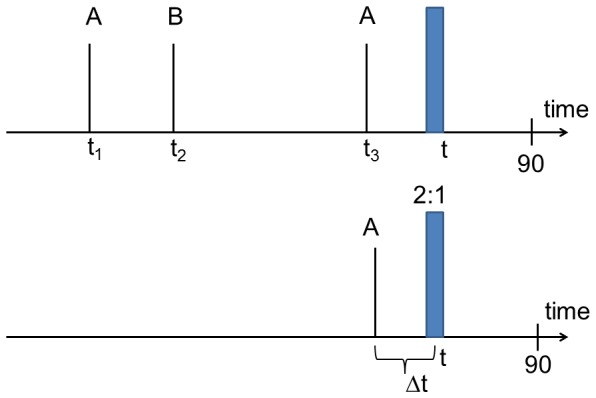
Scheme to describe the general statistical approach. Upper row: the complete information about the goals for a specific example which contribute to the prediction of the goal rate 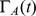 at time t. Lower row: the reduced information which takes into account the score at time t as well as the time difference 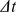 to the last goal and the information about the team which scored that goal.

An important first step is the *systematic* identification of the most relevant items for the conditioning of 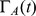. Let's assume that in minute 70 in total 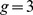 goals have been scored and that the actual score is 2∶1 for team A. The goals of team A were scored in minutes 10 and 60 and the goal of team B in minute 25. Strictly speaking we want to understand the impact of the previous goal events on the goal rate at a given time 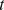. 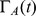 is thus conditioned on the sequence of the previous goals as well as the precise time of these goals. This is illustrated in the upper part of Fig.2. Apart from the fact that this complete dependence is impossible to extract from the available information one can dramatically simplify the required conditioning. For a specific example it will be shown that neither the order of the goals (e.g. 0∶1 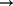 1∶1 vs. 1∶0 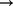 1∶1) nor the *absolute* times 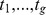 of the goals play a relevant role. We just mention in passing that the latter disagrees with the general belief that goals just before half time are particularly helpful for a team. These observations strongly suggest that in general the dependence on the order and the absolute times of the previous goals is, if existent at all, very weak. Thus, neglecting these pieces of information does not reduce the estimation quality of 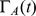. As a strict consequence 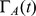 can only depend on the following observables: (1) Score in minute 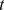. What is the expected course of the match during minute 70 if, e.g., the home team leads by one goal? In what follows we mainly restrict ourselves to the goal difference rather than to the absolute number of goals. (2) *Relative* time differences 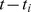 (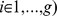. One may indeed expect that scoring a goal may give rise to a minor shock to the opponent which, as a consequence, may bias the match during the minutes after the goal. Naturally the impact of the last goal is strongest. Thus, we only keep track of the time difference 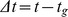. This reduction of information is summarized in the lower part of Fig.2. As soon as the goal rate depends on the present score one leaves the regime of Poisson processes and, in general, (possibly small) deviations from a strict Poisson goal distribution would be expected. Furthermore, any dependence on the time elapsed since the previous goal is a clear signature of non-Markovian effects since memory effects start to play a role.

## Results

### Total number of goals

We start with the discussion of the time dependence of the total number of goals in minute 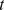, i.e. 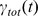. The data were already shown in Fig.1. Except for some specific minutes one observes a linear increase of the total goal rate with time. As compared to this linear trend the values in the 1st and the 46th minute are reduced by approx. 50%, respectively. This expresses the fact that the initial condition of the match (full separation of both teams in the halves of the field) implies a minimum time until the first goal can be scored. Roughly speaking, equilibration is reached after 30 seconds. The spikes in the 45th and 90th minute have the trivial origin that a match typically has some overtime which, however, is counted as minute 45 (after the first half) or as minute 90 (at the end). Somewhat surprisingly, no deviations from the linear trend are seen after the half time break (except for the obvious reduction of the goal rate at minute 46). This means that the match is basically continued as if there had not been any break. Furthermore a significant increase is observed beyond minute 87, representing an increasing offensive (or decreasing defensive) behavior.

The time-dependent rate still allows the whole process to be Poisson. Let us, for reasons of simplicity, consider the case where the average number of goals in the first half is 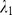 and in the second half 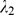. Then the probability to have 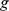 goals in the total match can be written as
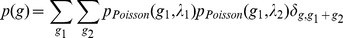
(5)where 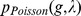 denotes the standard Poisson distribution. Application of the binomial equation yields after a straightforward calculation 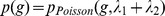. Thus, a time-dependent goal rate still allows the match outcome to fulfill Poisson statistics.

**Figure 3:**
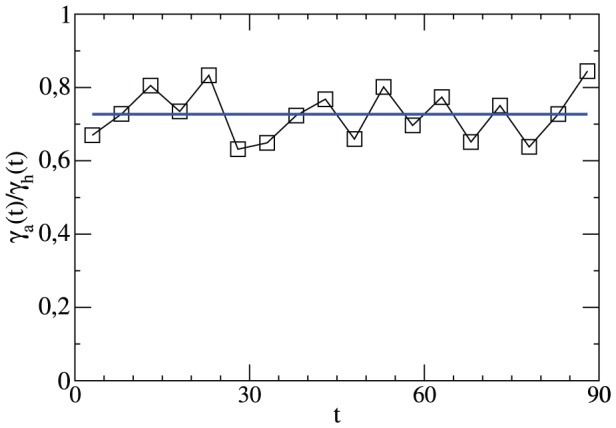
Average number of goals of home and away teams. Specifically shown is the ratio of the average number of goals of the away team vs. those of the home time as a function of the time t.

It may be instructive to analyze the ratio of goals, scored by the away team and the home team, respectively, as shown in Fig.3. It turns out that within fluctuations this ratio is constant throughout the match. Thus, no additional home-away-asymmetry has to be taken into account for the statistical description of the total goal rate.

### Dependence on the previous course of the match

For the study of a possible dependence on history we start by analyzing the dependence on the order of the previous goals. As a specific example we take all matches with the score 1∶1 at half time and check whether it makes a difference which team scored the first goal of the match. If the home team scores the first goal one obtains an average goal difference of 0.43 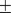 0.07 for the second half of the match. In the other case one obtains 0.46 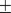 0.07. Within statistical uncertainty no difference can be observed so that in this specific case the order of goals plays no role. This motivates our choice to neglect the specific order of the previous goals in what follows.

Next we study a possible dependence on the exact time of a goal. Following the general ideas of Ref.[21] we record the outcome of the second half of a match under the condition that one team leads 1∶0 at half time. Under this condition the leading team will score on average approx. 0.15 goals more than the opponent in the second half. This observation just reflects the fact that the team, leading 1∶0, tends to be the favorite and thus more likely will be also more successful in the second half. As seen in Fig.? 4 and in agreement with the results of Ref.[21] the outcome of the second half is fully uncorrelated to the time of the first goal. Thus, a goal just before half time is no more influential on the further course of the match than an earlier goal. This observation suggests that it is not important to keep into account the absolute values of the scoring times. Thus, we omit the information about scoring times in order to strongly simplify the statistical description. Of course, strictly speaking a mild dependence cannot be excluded for other scores. Since testing all scores and all scoring times is impossible for statistical reasons we did not proceed any further with this question.

**Figure 4:**
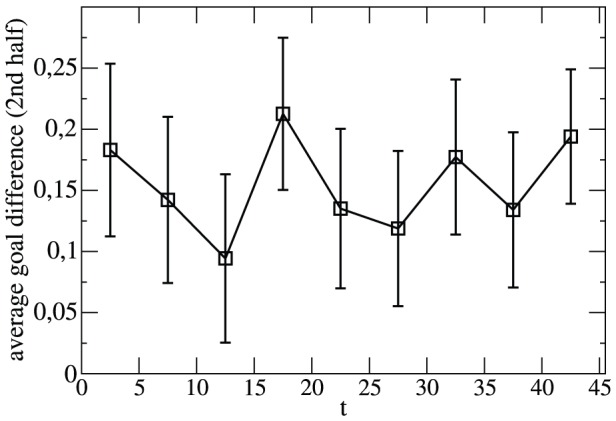
Dependence of the match b ehavior on the time of a goal. Shown is the goal difference in the second half of the team leading by 1∶0 at half time as a function of the time when this single goal has been scored.

**Figure 5:**
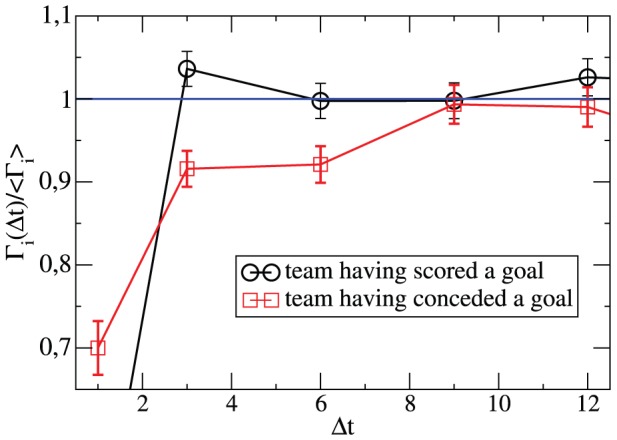
Dependence of the match behavior on the time elapsed since the previous goal. The goal rate of a team if a time 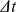 before it has scored or conceded a goal, respectively. The data have been normalized such that the average for 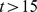 is unity. Except for 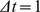 all data points result from an average over three minutes.

In the next step we study the time which passed since the last goal. Does scoring a goal change the pattern of the soccer match in the minutes after this goal? For this purpose we analyze the goal rate 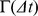 at time 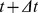 if at time 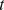 a goal had been scored. In analogy to our discussion in Sect. II we average over all instances where this condition is fulfilled. Here we distinguish whether the same team or the opponent had scored that goal (expressed by the index 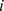 in Fig.5). The data are normalized by 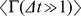, i.e. the typical goal rate sufficiently far away from the goal under consideration. In this way possible anomalies after scoring a goal can be directly read off for small 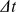. We start with the team which has scored the goal at time 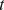. Naturally, at time 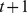 its goal rate is trivially suppressed because (a) there is a short break and (b) the opponent receives the ball. However, very soon the goal rate has reached its long-time limit. The data point at time 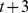 is slightly increased by approx. 3%. However, this minor increase may be related to statistical fluctuations. Thus, apart from the trivial short-time effect, scoring a goal has no significant impact for that team. Most interestingly, this is not true for the opposing team. Here the initial goal rate is suppressed by approx. 10% and the equilibration roughly takes nearly 10 minutes. Thus, after a goal this team is less active for a couple of minutes. This more defensive attitude, however, does not reduce the number of conceded goals. Again there is an anomaly for minute 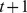 because of the break after the goal.

In summary, apart from the minor short-time effects as displayed in Fig.5 no memory effects are present. Stated differently: playing soccer is to a large extent a Markovian process, i.e. the action during the next minute does not depend on the time-history of how the present score has been generated.

### Dependence on the present score

**Figure 6:**
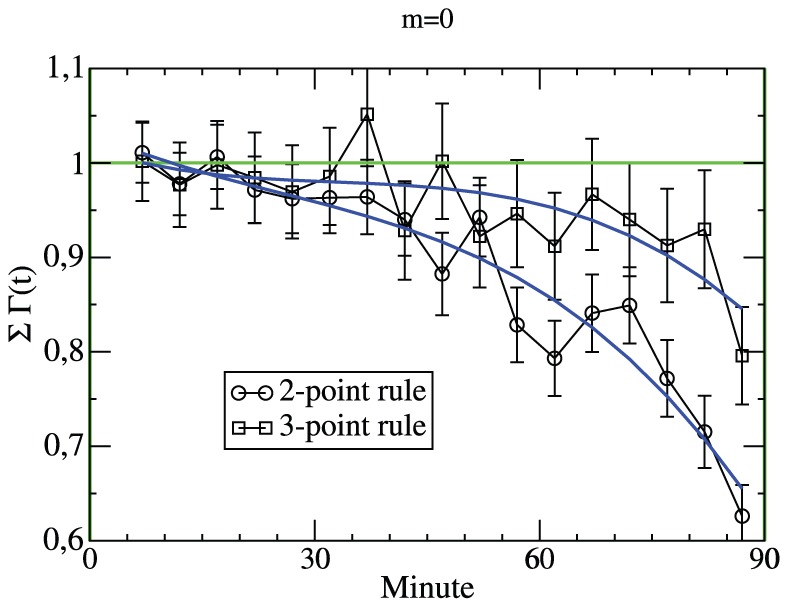
Total goal rate for the score of 0∶0. The normalized number of goals in minute 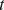 if the score is 0∶0. Distinguished are the seasons with the 2-point rule and with the 3-point rule, i.e. before and after 1995.

In the remaining part of this work we analyze the question whether the present score has any influence on the goal rate. We start by analyzing the total goal rate, i.e. 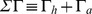 at time 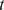 when the score is 0∶0; see Fig.6. Starting already from the middle of the first half of the match the total goal rate tends to decrease. During the last five minutes the rate is nearly 40% smaller than the average rate. Actually this value was observed for the seasons until 1994/95. Afterwards the 3-point rule has been introduced, rendering the draw more unfavorable. Indeed the effect became less relevant but is still significant (approx. 20% reduction during the last 5 minutes). Thus, the coziness of a draw is still present in the heads of the soccer players.

**Figure 7:**
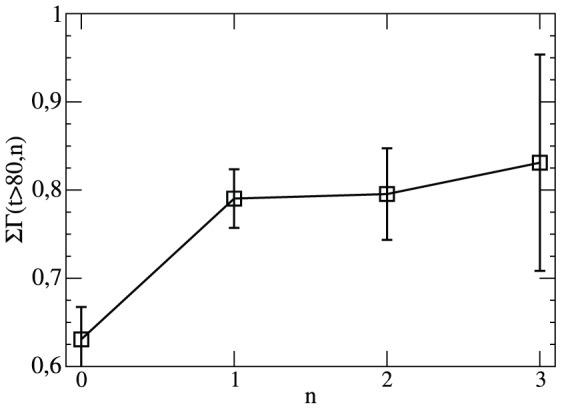
Total goal rate in the final 10 minutes for different ties. The normalized number of goals in the last 10 minutes under the condition that at minute 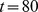 the score is 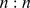.

Actually, this effect is particularly pronounced in case of a 0∶0. This can be seen from Fig.7 where we have plotted the reduction in the total goal rate vs. the score (0∶0,1∶1,etc.) during the last ten minutes. More specifically, we have calculated the goal rate at time 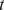 if the score is 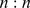 and then averaged this rate over 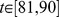. It turns out that for all different types of draws the players seem to be happy with the result, even in case of a 3∶3 where already 6 goals had been scored. In summary, we have found the first example where the match behavior significantly depends on the present score.

Of course, as an immediate consequence the total goal rate in case of a non-zero goal difference has to increase beyond unity (approx. 1.07) because by definition the average over all matches has to be unity.

**Figure 8:**
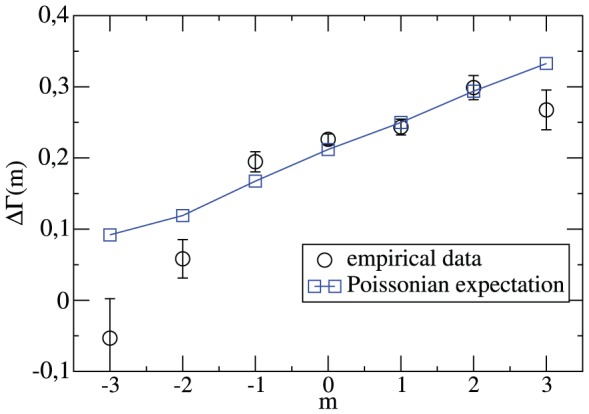
Relative goal rate for different scores. The normalized difference of goal rates if the goal difference is 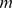 Included is the Poisson expectation.

More interesting than the sum of the goal rates is the difference as expressed by 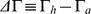. It contains the information whether the balance between both teams in a match is disturbed due to the present score. Specifically, we consider 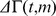, i.e. the dependence on time and goal difference. In a first step we average 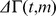 over all times with weighting factors which take into account how often a specific score is present at time 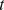. The resulting observable is denoted 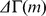. One might speculate that a team which is far ahead has an excellent day and very likely will further increase its lead in the near future. Indeed, we find in Fig.8 a monotonous increase of 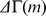 with the value of 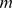. The fact that for all values of 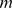 except for 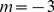 this difference is positive just represents the omnipresent home advantage.

There are two extreme cases to explain this behavior. First, there exist psychological effects which may render sportsmen more successful in case of a lead. This effect would go beyond the simple Poisson scenario and complicate the statistical description of soccer matches. Second, it is a simple selection effect. Teams, leading by three goals simply tend to be the better team and therefore also score more goals in the future. In contrast to the first scenario the future success is thus already determined before the match and just reflects the different team strengths.

We can check whether the fitness variation among different teams can fully explain the observed behavior. For this purpose we additionally estimate the probability that the home or the away team will score a goal in minute 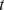 for the case of simple Poisson statistics. According to Sect.2 we simply estimate 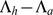 for the corresponding matches. In this way we may take into account the above-mentioned selection effects. These Poisson estimations are also included in Fig.8. One can clearly see that to a large extent the increase of 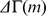 with 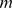 can be explained by the selection effect. Thus, self-affirmative processes [7], [8], [11] are not required to explain why with a score of 2∶0 the home team will be more successful in the next minute as compared to a 0∶2 situation. More generally, to a first approximation the present score of the match does not influence the relative success of both teams in the near future.

However, a more detailed analysis reveals some deviations from the simple Poisson estimation. For 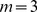 there seems to be some type of saturation mechanism which slightly reduces the tendency to further increase the goal difference. Similarly, for 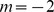 or 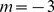 the home team has a tendency to resign.

**Figure 9:**
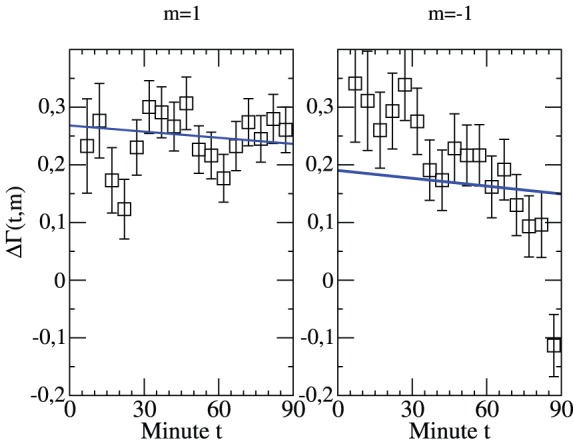
Time-resolved relative goal rate for a one-goal lead. The normalized difference of goal rates in minute 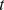 under the condition that either the home team (left, 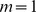) or the away team (right, 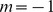) leads by one goal. Included is the Poisson expectation.

In analogy to Fig.6 we now present the time-resolved rates in Fig.9. We compare the cases where either the home team or the away team leads by one goal in minute 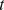. We start the discussion with 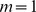. Evidently, it is more likely in the next minute to increase the one-goal lead rather than to reach a draw. This is mainly an effect of the home advantage. To first approximation the tendency towards an increasing lead is independent of the time of the match. Furthermore, its value is fully consistent with the Poisson expectation. We may conclude that in case of a one-goal lead of the home team the match behavior follows a simple Poisson behavior.

A closer inspection of 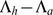 shows that this value somewhat decreases with increasing time. This has a simple interpretation. If the first goal of the home team is already scored after 10 minutes it is more likely that the home team is by far the stronger team. Due to the highly random nature of a soccer match this effect is weak and the discussion of Fig.6 is not influenced by the weak time-dependence of 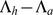.

Most interestingly, the situation is very different if the away team leads by one goal as can be also seen from Fig.9. One may distinguish three time regimes. In case of an early lead (t 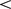 35) of the away team the home team is more successful than expected from the Poisson expectation to equal the score. The increase of 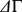 as compared to the Poisson expectation is as large as 50%. For t 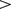 35 the course of the match behaves as expected from the statistical behavior. However, for t 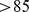 a dramatic change is observed. Suddenly it becomes even more likely that the away team scores the second goal as compared to a draw.

In order to clarify the strong decay of 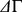 during the last minutes of a match we have individually determined 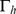 and 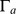; see Fig.? 10. One can clearly see that the anomalies at the end of the match are exclusively related to 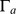, i.e. the offensive of the away team and the defensive of the home team. During the last 5–10 minutes the defensive of the home team becomes much weaker. A straightforward interpretation of this observation is the strengthening of the offensive efforts at the expense of the defense. Unfortunately, on average these attempts are in vain because the only effect is a larger number of conceded goals. In the most extreme variant of this endeavor even the goal keeper starts to support the own strikers. In any event, this behavior contributes to the increase of 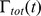 in the last minutes of the match as discussed in Fig.1.

**Figure 10:**
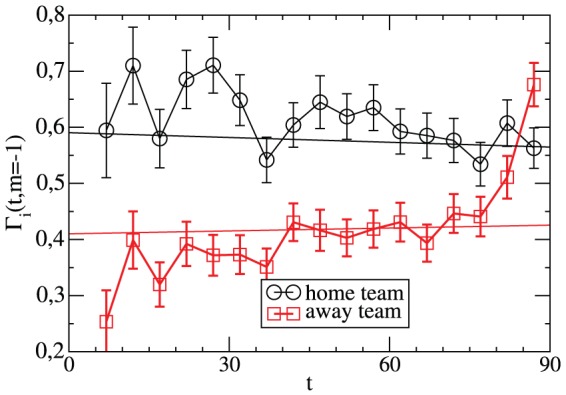
Time-resolved goal rates for both teams for a one-goal lead of the away team. The normalized number of goals in minute 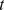 of the home and away team, respectively, under the condition that the away team leads by one goal. Included is the Poisson expectation.

The deviations from simple Poisson behavior for short times is both related to an increase of 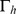 and a decrease of 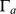. Obviously, in the first half of the match the home team is still able in a focused and successful manner to intensify its effort to reach a draw with an improvement in the offensive and defensive part.

**Figure 11:**
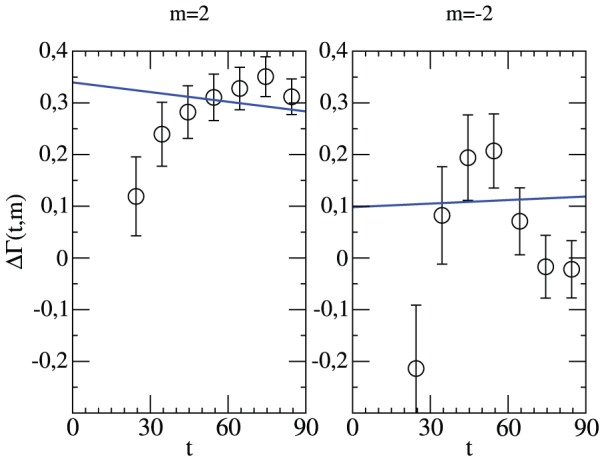
Time-resolved relative goal rate for a two-goal lead. The normalized goal difference in minute 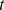 under the condition that either the home team (left, 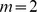) or the away team (right, 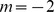) leads by two goals. Included is the Poisson expectation.

We have repeated this analysis for 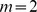 and 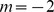; see Fig.11. Here some additional effects emerge. If the home team leads by two goals very early in the match (around minute 20 to 30), the superiority is smaller than expected from the Poisson expectation. Starting from minute 40 a similar behavior is observed as for 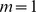. In the opposite case 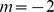 one observes that an early two-goal lag has dramatic consequences on the performance of the home team. 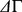 is strongly reduced so that it is even more likely that the away team scores the next goal. Note that the Poisson expectation would still predict a small but significant home advantage. Only between minutes 40 and 60 the home team successfully attemps to reduce a two-goal lag. Starting from minute 60 these attemps start to be in vain.

## Discussion and Summary

Based on our systematic approach to identify deviations either from the the Poisson expectation and/or from a strict Markovian behavior we have obtained several key effects to characterize complexities of soccer matches. (1) After a goal the opponent is less successful to score a goal during the next minutes. This invalidates a strict Markovian picture of soccer matches. This effect, albeit significant, is relatively small (10%). (2) In case of a draw the total goal rate becomes smaller. Thus the goal rates have to be adjusted in dependence of time and score. This is a strong deviation from the Poisson expectation. (3) In case of a lead of the away team dramatic deviations from the Poisson expectation are observed during the last 5–10 minutes of the match. This effect reflects inefficient defensive behavior of the home team. The latter point indicates a dramatic difference of the behavior of home and away teams which goes beyond the mere home advantage. It signals strong psychological and/or tactical differences for home as compared to away teams. Since the offensive efficiency does not become worse the present result does not imply that the home team gives up, at least in case of a one-goal lead by the away team. (4) If, however, the lead of the away team occurs in the middle of the match there are indications of an improved efficiency of the home team to equalize.

With respect to (1) it is interesting to refer to recent work on scoring events in basketball. It has successfully been described in terms of a biased continuous time random walk [17]. Ideally the time difference between successive scoring events should follow an exponential distribution. In practice already 20 seconds after a score the actual data follow very well this theoretical expectation. In contrast, in the field of tennis statistics significant deviations from purely statistical behavior have been observed by Magnus and Klaassen [18]. For example, after a break point it is more likely to win the next service game. Interestingly, this effect is more pronounced in matches between non-seeded players. This indicates that with increasing quality of the players the impact of previous effects become smaller, i.e. the match follows more a Markovian behaviour.

Our results also allow us to find an answer to our initial question about the origin of the large number of draws. It is the persistence of a draw, i.e. (2), rather than the ability of a team, trailing by one goal, to score an additional goal as expressed by (3). Actually, (3) would rather decrease the number of draws because the probability that a 0∶1 transforms in a 0∶2 during the last minutes is significantly larger than expected.

However, we should also stress that at least during the first 80 minutes most observables behave according to the simple Poisson expectation as expressed by Eq. 4. This observation may be used to discuss an important general question. Does the empirical observation of a nearly Poisson-type goal distribution imply that the process of scoring goals is indeed characterized by some fixed rates? Alternatively one might postulate that good teams try to achieve a safe lead and then just start to manage the lead. In this scenario our in-match analysis should have detected much larger deviations from Poisson behavior. For example one might have guessed that 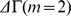 is much smaller than expected from the Poisson scenario. Since this is not observed, the teams typically do not change their match behavior. Differences along this line just start to (slightly) occur for 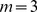.

In summary, we may conclude that the concept of score-insensitive goal rates as opposed to score-dependent match behavior is a very good approximation of a soccer match, at least after averaging over the corresponding subset of matches as done in this work. This naturally explains the previous observation [10], [14]–[16] that the goal distribution, after taking into account the different team strengths, follows very nicely a Poisson distribution. This conclusion has an interesting consequence. A match of a good team and bad team may have a priori goal expectations of 2 and 1, respectively. A specific Poisson realization may, e.g., lead to a 3∶0 or (more unlikely) to a 1∶3 result. In both realizations the quality of the good team and that of the bad team are identical because the final result is just a matter of mere luck (in analogy to the presence or absence of the decay of a radioactive nucleus during a fixed time interval). In practice, one might expect that in the first case media stress the successful play of the favorite whereas in the second case the same team would be strongly criticized. This reaction would neglected the random aspects, inherent in any Poisson realization and just show that an objective assessment of random aspects is very difficult.

It may be interesting in future work to check whether, e.g., the subset of good teams is less sensitive to negative effects (having just conceded a goal, leading behind at the end of the match). The present results may then serve as a detailed basis for the identification of possible strength-dependent effects.
